# Appendicitis

**Published:** 1890-12

**Authors:** A. S. McDaniel

**Affiliations:** San Antonio


For Daniel's Texas Medical Journal.

### APPENDICITIS.

BY A. S. M'DANIEL, M. D., SAN ANTONIO.

Read at the West Texas Medical Association meeting October 31, 1890.

I WISH to present to-day for your consideration and discussion a comparatively new surgical procedure.

I allude to the inflammation, ulceration and perforation of the appendix vermiformis, and operation for same.

The appendix is, as you all well know, situated at the lower and back part of caput cæcum coli, is three to six inches in length, diameter about that of a goose quill. Its canal is small and communicates with the cæcum by an orifice, guarded by a valve. The appendix is, of all organs of males, in the abdomen, most prone to dangerous inflammations. In females this tendency is exceeded by the pelvic organs alone.

The appendix is fourfold more liable to inflammation in males than females.

The great majority of cases of so called typhlitis and perityphlitis begins as appendicitis. The tissue of and about the cæcum being inflamed secondarily. Where cæcitis appears primarily, it is exceptional for it to lead to ulceration and perforation. Where the cæcum is perforated, it is from without, and due to pressure of an abscess, the result of an appendicitis.

The failure of free evacuation of the appendix is usually the first step towards inflammation. Filled with enteroliths, cherry-seed, grape-seed, with mucus, and products of decomposition, it becomes inflamed, then undergoes ulceration of the mucous lining, which may produce perforation, resulting in peritonitis, or cellulitis, depending on the location of the opening. If peritonitis, a localized inflammation, and probably an intra-peritoneal abscess. Most cases in males of so-called inflammation of the bowels are instances of inflammation of the appendix.

Most cases of peritonitis in male patients are due to perforation or inflammation of this organ. Many cases of supposed intestinal obstruction are instances of ulceration and perforation of the appendix. Abscesses rarely occur till after perforation.

The fatality of all cases of appendicitis is not great. The fatality after perforation is however very great. The deaths are caused from peritonitis, from wasting discharges from the abscess, and septicemia.

One-third of all post-mortems show signs of old inflammations of the appendix.

I, myself, after becoming interested in this subject, noticed the bodies in the dissecting-room and dead-house of Bellevue College, and was surprised to find evidence not only of previous inflammations, but many ulcerated and perforated appendices.

The diagnosis of perityphlitis is usually easy, as there is always a tumor over the cæcum.

The diagnosis of appendicitis is however, more difficult, since there is no tumor, the tenderness is often slight and very deep.

The diagnosis must be made largely by exclusion.

Acute appendicitis may be ushered in by vomiting and pain in the epigastrium, which may mask pain in the cæcal region.

A large portion of first attacks, and many after attacks, will recover without abscess, under proper treatment, quiescence in bed, hot applications, anodynes, opium principally, light diet and avoidance of all cathartics, and all influences causing peristalsis. Reliance on treatment is justifiable in the absence of evidence of perforation, abscess, induration, lasting two or three days, or of protracted severe pain, resulting in high temperature.

A few cases will suffer sudden perforation, general peritonitis, and death, but nearly all of them failing to recover promptly will, if perforation occur, have sharply localized peritonitis, or abscess, which will be readily recognized.

Surgical interference is demanded:

1. In cases of protracted high temperature and distinct induration, that does not yield in forty-eight hours.

2. In cases of severe inflammation, lasting several days, even though no induration can be made out, and in localized peritonitis, having its origin in the appendix and producing evidence of abscess.

3. In all cases where large indurations develop rapidly, with high fever; here extensive deposit and abscess are almost certain, and the operation should be early.

4. In all cases advanced to subacute, or chronic appendicitis, with induration, and dullness, regardless of temperature.

5. In all undoubted chronic cases of appendicitis, recurring exacerbations, whether induration can be made out or not. In all such cases, the patient is in constant danger of perforation and fatal peritonitis, and extirpation of the organ, if done antiseptically, reduces the hazard.

When to operate the most is important question. It used to be thought not proper to operate until adhesion had formed, but we now know that while we are waiting, perforation may occur and the patient die.

The local condition of the parts should be considered. There should be a certain amount of redness and a certain amount of swelling present.

Œdema is a certain indication of pus, and this should exist before you operate.

You should also have a suppurative chart, the temperature going up to one hundred and three in the evening, and down to one hundred and one in the morning. If the fever goes to one hundred and four, and drops to normal, this would be from sympathetic nervous system, and would not indicate pus. The pus chart never goes to normal.

How long must we wait when this condition is present? After the eighth day seventeen per cent. die; before the eight day eight per cent.; consequently the operation should be done before the eighth day.

### THE OPERATION.

We should remember, that in these cases, we have a sac formed, and circumscribed peritonitis, this being the only safeguard the patient has, and therefore, we must be very careful, lest we open the peritoneal cavity. Make the incision, which should be about four inches in length, so as to open the sac. Begin at the center, and two inches above Poupart's ligament, carrying it upward and outward, so the center will be opposite the anterior superior spine of ilium, going carefully through the integument and fascia and the aponeurosis of external oblique, and internal oblique, and transversalis muscle. Then stop and be very careful, for with the next cut, you kill or cure. When the edges of the wound are pulled apart, feel for fluctuation, and keep close to the ilium. You will find sac formed from lymph, and in this sac you will find the cuniform appendix. Many other things are also frequently found in this sac, gall-stones, cherry-seed, pieces of oyster shells, etc.

When you find the appendix, you should pull up the cæcum and excise the appendix, and sew the edges together with continuous catgut suture. Gently wash out the cavity, and insert drainage tube, or pack with iodoform gause, if there is much pus.

